# Potential Applications of Imaging and Image-Guided Radiotherapy for Brain Metastases and Glioblastoma to Improve Patient Quality of Life

**DOI:** 10.3389/fonc.2013.00284

**Published:** 2013-11-19

**Authors:** Nam P. Nguyen, Mai L. Nguyen, Jacqueline Vock, Claire Lemanski, Christine Kerr, Vincent Vinh-Hung, Alexander Chi, Rihan Khan, William Woods, Gabor Altdorfer, Mark D’Andrea, Ulf Karlsson, Russ Hamilton, Fred Ampil

**Affiliations:** ^1^Department of Radiation Oncology, The University of Arizona, Tucson, AZ, USA; ^2^Department of Psychology, Stanford University, Palo Alto, CA, USA; ^3^Department of Radiation Oncology, Lindenhofspital, Bern, Switzerland; ^4^Department of Radiation Oncology, Centre Val d’Aurelle, Montpellier, France; ^5^Department of Radiation Oncology, University Hospitals of Geneva, Geneva, Switzerland; ^6^Department of Radiation Oncology, University of West Virginia, Morgantown, WV, USA; ^7^Department of Radiology, The University of Arizona, Tucson, AZ, USA; ^8^Department of Radiation Oncology, Richard A. Henson Cancer Institute, Salisbury, ML, USA; ^9^Department of Radiation Oncology, Camden Clark Cancer Center, Parkersburg, WV, USA; ^10^Department of Radiation Oncology, Southeast Treatment Center, Houston, TX, USA; ^11^Department of Radiation Oncology, Marshfield Clinic, Marshfield, WI, USA; ^12^Department of Radiation Oncology, Louisiana State University, Shreveport, LA, USA

**Keywords:** glioblastoma, brain metastases, image-guided radiotherapy, neurotoxicity

## Abstract

Treatment of glioblastoma multiforme (GBM) and brain metastasis remains a challenge because of the poor survival and the potential for brain damage following radiation. Despite concurrent chemotherapy and radiation dose escalation, local recurrence remains the predominant pattern of failure in GBM most likely secondary to repopulation of cancer stem cells. Even though radiotherapy is highly effective for local control of radio-resistant tumors such as melanoma and renal cell cancer, systemic disease progression is the cause of death in most patients with brain metastasis. Preservation of quality of life (QOL) of cancer survivors is the main issue for patients with brain metastasis. Image-guided radiotherapy (IGRT) by virtue of precise radiation dose delivery may reduce treatment time of patients with GBM without excessive toxicity and potentially improve neurocognitive function with preservation of local control in patients with brain metastasis. Future prospective trials for primary brain tumors or brain metastasis should include IGRT to assess its efficacy to improve patient QOL.

## Introduction

Treatment of glioblastoma multiforme (GBM) and brain metastasis remains a challenge because the poor survival and potential for brain damage. Despite the fact that GBM is a primary glioma and brain metastasis represents dissemination to the brain of solid malignant tumors, median survival for both conditions remains similar because of the lack of treatment efficacy. Prognosis factors for both GBM and brain metastasis are age and performance status based on the recursive partitioning analysis (1, 2). Thus, given the remote chance for long-term survival, the goal of treatment should be the improvement of quality of life (QOL) for GBM and brain metastasis patients. New radiotherapy techniques such as image-guided radiotherapy (IGRT) provides the clinician with an unique opportunity to shorten the length of treatment without increasing treatment toxicity by virtue of normal tissue sparing. Magnetic resonance imaging (MRI) is now integrated into of radiotherapy planning to improve treatment accuracy. New imaging modalities such as positron emission tomography (PET) and diffusion tensor imaging (DTI) may also accurately delineate the gross tumor volume (GTV) and the areas of radiation brain damage respectively, and complement MRI to decrease normal tissue toxicity. In the following sections, we will review the literature to determine how to best combine new diagnostic technology with advanced radiation treatment to improve patient QOL.

## Treatment of Glioblastoma

Standard treatment for GBM is surgical resection if feasible followed by concurrent chemotherapy with temozolomide (TMZ) and radiation (3). Despite the addition of TMZ, only 9.8% of the patients survived at 5 years. The predominant pattern of recurrence is local failure suggesting that improving local control may improve survival. Many institutions have attempted to increase radiation dose to the tumor bed for better local control. However, a randomized study using radiosurgery as a boost dose to the tumor bed prior to chemoradiation did not demonstrate any improvement of survival. Two hundred and three patients with supratentorial GBM were randomized between carmustine (BCNU) and standard radiation to 60 Gy or radiosurgery boost followed by standard radiation. Local failures occurred in 90% for both groups causing patient death (4). These clinical results suggest that GBM is both chemo- and radio-resistant. Recent studies in molecular biology have demonstrated the existence of cancer stem cells (CSCs) in GBM responsible for treatment failure. Despite high doses of radiation of 30–60 Gy in a single fraction, these CSCs continued to proliferate in cell cultures following radiation (5). The radio-resistance of GBM cells suggests that radiation dose escalation alone is not feasible to control tumor growth in the clinical setting because of the excessive neurotoxicity associated with such a high dose. The mechanism of chemo-resistance of CSCs is complex but one of the principal mechanisms is the presence of transporters that actively pump the drugs out of the cells preventing their tumoricidal actions (6). Thus, unless new therapies are directed toward the control of CSCs, conventional postoperative chemotherapy and radiation is doomed to fail. A reasonable alternative to the standard fractionation schedule would be shortening the course of radiotherapy which may allow the patient to spend quality time with their family provided that the accelerated course does not increase toxicity. A randomized trial of TMZ versus standard radiotherapy versus hypo-fractionated radiotherapy in elderly patients showed a better survival in patients above 70 years treated by hypo-fractionated radiotherapy or TMZ as compared to standard radiotherapy (7).

## Treatment of Brain Metastases

Even though the optimal treatment for brain metastasis remains controversial, radiation therapy is very effective to prevent disease progression even for radio-resistant tumors such as renal cell cancer and melanoma. The delivery of a very high radiation dose with radiosurgery provides excellent local control (8). However, when there are multiple metastases or when there is evidence of tumor progression after radiosurgery, whole brain radiotherapy (WBRT) is required. In patients who had tumor regression following whole brain irradiation, neurocognitive function, and survival improve suggesting that local control of the tumor in the brain remains the most important factor to prevent deterioration of mental status (9). Even though ultimately all patients experienced deterioration of their neurocognitive function following radiosurgery alone or radiosurgery with WBRT, improvement of local control with the addition of WBRT delayed the time to deterioration emphasizing the importance of local control on mental status (10). There are still controversies whether WBRT should be added to radiosurgery for brain metastasis at initial diagnosis. In one study, the poor survival observed with WBRT and radiosurgery compared to radiosurgery alone is most likely due to the delay to initiate chemotherapy in patients who received WBRT (11). Most of the patients treated for brain metastasis ultimately died from systemic disease progression emphasizing the fact that while local control remains important, chemotherapy remains the main treatment and should not be delayed unnecessarily (12). Thus, future studies should focus on decreasing the overall treatment time and the neurotoxicity of WBRT while optimizing local control of brain metastases.

## Radiation-Induced Neurotoxicity

Animal experiments demonstrate normal brain injury following WBRT. Adult rats exposed to single fraction whole brain irradiation to 25 Gy developed decreased cognitive function compared to sham-irradiated rats (13). Autopsy of the irradiated rat brains revealed demyelination with or without necrosis mainly in the corpus callosum. Increased gliosis was also observed similar to the one reported in human brains affected by accelerated aging such as Alzheimer’s disease and multiple sclerosis. Damage to the normal brain is dose-dependent as adults rats exposed to whole brain fractionated irradiation to 30 Gy in 10 fractions developed memory loss without observed microscopic damage (14). The mechanism of brain injury at the molecular level is complex and is postulated secondary to depletion of oligodendrocytes leading to demyelination, deletion of neural stem cells (NSCs) in the hippocampus, cerebral cortex, vascular injury, and more recently vasculitis (15, 16). The use of MRI-based techniques such as DTI allows for monitoring of human brain damage following irradiation. DTI measures water molecule diffusion in the brain which varies with the direction, density, and myelination of white matter fibers. Diffusion of water perpendicular and parallel to white fibers is termed radial diffusivity (RD) and axial diffusivity (AD) respectively. Increased RD and decreased AD has been correlated to decreased myelination and increased gliosis respectively. Using DTI prior to and 1 month post WBRT in 14 patients with brain metastases, increased RD was observed in all brain structures but more prominent in the cingula and fornix suggesting demyelination of the limbic structures responsible for memory and behavior (17). Radiation-induced demyelination of the white matter tract was also corroborated in another study also showing a heterogeneous extent of injury despite a uniform radiation dose suggesting that some white matter tracts are more sensitive than others (18). An autopsy case report of a patient dying following radiation myelopathy also demonstrated extensive demyelination and axonal loss without vascular damage corroborating the DTI report (19). In addition, WBRT or partial brain irradiation damages the NSCs which usually remain dormant within the subventricular zone. Following a moderate radiation dose of 4 Gy, NSCs start to proliferate exposing them to death from apoptosis with higher radiation doses (20, 21). Animal experiments demonstrated a direct relationship between radiation damage to NSCs and neurocognitive dysfunction. Rats receiving WBRT developed cognitive dysfunction but if they were transplanted with NSCs in the hippocampus after radiation, the ones who received NSCs recovered their cognitive function compared to the ones who had sham surgery (22). The transplanted NSCs migrated extensively and differentiated into glial and neuronal lineages of the rat brain even though they were from human species suggesting that 1 day, human NSC transplants may be used to treat neurocognitive damage following brain irradiation (22). In another mouse model delivering a high radiation dose to the whole brain (20 Gy in 4 Gy/fraction) similar to the clinical whole brain treatment, the mice developed short term memory loss associated with decreased granular layer of the dentate gyrus of the hippocampus. However, if the irradiated mice received NSCs administered intravenously following each radiation treatment, they preserved both brain structure and function (23). Autopsy of patients who had WBRT also demonstrated depletion of neuronal cells in the hippocampus (24). Thus, protecting the limbic system from excessive radiation may reduce neurocognitive damage following WBRT.

## Current Impact of MRI on Radiotherapy Treatment Planning

Because of the non-ionizing technique that uses a strong magnetic field to provide high resolution anatomic information, MRI is now integrated into the radiotherapy planning for brain tumors. The accuracy of MRI to demonstrate tumor invasion of the normal organs has led to the development of MRI-based linear accelerators. Institutional preference dictates the choice of either T2-weighted MRI or FLAIR MRI to outline the GTV and surrounding edema as CTV. Traditionally T2-weighted MRI has been used as GTV delineation because biopsy of the area of MRI T2 abnormality demonstrated tumor cells outside of contrast enhanced CT abnormality. However, T2 weighting causes cranial spinal fluid (CSF) to be brighter which may potentially impair visualization of the GTV. The FLAIR sequence nullifies the CSF signal and may provide better GTV delineation. An expansion of 2 cm of the CTV is used to outline PTV. The FLAIR PTV is usually larger than the T2 PTV and may potentially increase normal tissue toxicity (25). On the other hand FLAIR images provides better tumor-to-CSF contrast compared to T2 and T1 weighted sequences and may be valuable for stereotactic planning of brain gliomas and metastases (26). Thus, incorporating FLAIR sequence into radiotherapy planning may potentially improve tumor targeting for radiation dose escalation.

The introduction of higher field strength MRI (3.0 T) (3T MRI) compared to the conventional 1.5 T (1.5T MRI) may potentially increase radiotherapy delivery accuracy because of higher image resolution. In a study of 138 patients with brain metastases, 22% were found to have a higher number of metastases with 3 T MRI compared to 1.5 T MRI. All patients were treated with radiosurgery with the radiotherapy planning based on 3 T MRI and would have had geographic miss if 1.5 T MRI was used for treatment planning (27). Patients with multiple brain metastases are more likely to have additional lesions seen on 3 T MRI (28). The superiority of 3 T MRI for radiosurgery planning compared to 1.5 T MRI was also corroborated in another study (29). Thus, even though these studies are only preliminary, 3 T MRI may have an increasing importance in the future for radiotherapy planning.

## Potential Role of *O*-(2-[^18^F]-Fluoroethyl-l-Tyrosine Positron Emission Tomography in Radiotherapy Planning and Treatment of GBM

Accurate tumor delineation is the first step in radiotherapy planning to avoid marginal miss and to decrease excessive radiation dose to the critical structures adjacent to the tumor. Standard imaging for neurologic oncology has been MRI with gadolinium contrast. The extent of contrast enhancement on MRI is used to determine the GTV or as an indicator of therapeutic response. However, contrast enhancement due to the transient blood brain barrier breakdown following surgery, may mimic tumor progression and interfere with the GTV delineation. *O*-(2-[^18^F]-fluoroethyl-l-tyrosine (FET) is an amino acid analog radiolabeled with fluorine 18. After crossing the blood brain barrier, FET is taken by LAT2 transporters located on the membranes of the GBM cells. Thus, high uptake of FET by the tumor cells allows for better visualization of the tumor compared to the normal brain. The advantages of FET include its long half-life (110 min), its ease for synthesis, its fast brain and tumor uptake kinetics, and low accumulation in non-tumor tissues making this radiotracer an ideal imaging technique in the outpatient setting (30). Because the tumor uptake of FET is independent of blood brain barrier disruption, FET-PET may be complementary to MRI to outline the exact extension of the tumor and serve as functional imaging for IGRT. In a study of 17 patients with biopsy proven GBM, FET-PET was compared to MRI for GTV delineation. The GTV based on FET-PET was larger in 10 patients, smaller in three, and the same in the remaining four (31). Perhaps, the major advantage of FET-PET over conventional MRI for radiotherapy planning is its ability to detect areas of high tumor activity within the GTV which manifest as a high standard uptake value (SUV) (32). These high SUV areas can be targeted with a higher radiation dose compared to the dose delivered to the GTV, thus potentially increasing tumor control without increasing radiation dose to the normal brain tissue. The main weaknesses of the clinical studies which failed to demonstrate a survival benefit for dose escalation in GBM are their reliance on MRI for target delineation and the radiotherapy technique employed which delivered a uniform dose across the GTV. The tumor concentration within the GTV is heterogeneous and areas with high concentration of actively dividing tumor cells may have residual tumor cells after radiation. On the other hand, increasing tumor dose to improve local control may lead to severe complications because of excessive irradiation of the adjacent normal brain tissue. Thus, a radiation technique that allows radiation dose escalation within the tumor without increasing the dose to the normal brain would be ideal. Integrating FET-PET into IGRT planning may be a solution to avoid neurotoxicity. As an illustration, the dosimetric advantage of IGRT with the simultaneous integrated boost (SIB) technique to spare the normal brain compared to the conventional sequential (SEQ) boost technique for primary brain tumors was reported recently (33). Other advantages of FET-PET are its better accuracy compared to MRI allowing better local control because of decreased risk of marginal miss and better ability to detect tumor after radiation (31, 34). FET-PET will likely play a prominent role in future IGRT studies for brain tumors.

## Technologies of IGRT Delivery

There are currently two systems for IGRT delivery which are grouped into radiation-based (kV and MV) and non-radiation based (ultrasound, electromagnetic) (35). Visualization of the tumor is either direct or through fiducial markers inserted into the tumor. The images acquired before the treatment are then compared to the ones acquired during radiotherapy planning. A shift in patient position is performed if there is any discrepancy in the set up and another set of images is obtained to verify treatment accuracy. Thus, daily imaging minimizes the risk for marginal miss due to positioning and patient movement during treatment. It is unclear which imaging modality is optimal for IGRT delivery. The choice of the IGRT technology most likely depends on clinician preference, the types of tumors most commonly treated at the radiation oncology institution, and budget constraints.

In the radiation based system, the image acquired prior to treatment are either 2-dimensional (2D) or 3-dimensional (3D). The quality of image is superior with kV imaging compared to MV imaging. Image acquisition in the 2D system relies on electronic portal imaging devices (EPIDs) where the treatment beam is captured on a flat panel behind the patient (Clinac, Elekta Oncology Primus) and stereotactic imaging. The stereotactic imaging relies on two kV X-ray sources mounted on ceiling or floor which provides orthogonal images and real time imaging (CyberKnife, Novalis TX, BrainLab). Even though the image quality is excellent, stereotactic imaging relies on bony landmarks or surrogate markers and does not provide soft tissue information. The kVCT (fan beam) imaging uses a diagnostic CT scan along side the linear accelerator (CT on rails, Siemens Medical Systems; ExaCT, Varian Medical Systems). Soft tissue information is excellent with the kVCT fan beam but the couch needs to be displaced between imaging and treatment which may lead to positioning error. The kVCT (cone beam) uses an gantry mounted kV source and a flat panel detector. A series of kV X-rays are taken when the gantry rotates and a 3D image is reconstructed. Even though the soft tissue special resolution is good, the image quality is inferior to fan beam kV CT (Synergy, Elekta; On Board Imager, Varian Medical Systems; Artiste, Siemens Medical Systems). In the MV CT fan beam system, the imaging is performed by the treatment beam which rotates around the patient while the couch moves (Helical Tomotherapy). There is no metal artifacts but the image quality is inferior to kV CT.

In the non-radiation based system, an ultrasound is performed before the treatment for target localization (usually prostate). The system is simple, non-invasive, inexpensive but operator dependent (Varian Medical Systems, B Mode Acquisition, and Targeting; Nomos, Elekta Oncology). Another non-radiation based system relies on the implantation of electromagnetic transponders inserted in the target (usually prostate) which provides tracking of the tumor motion (Calypso). The last non-radiation based system which is just approved by the US Food and Drug Administration involves a hybrid MRI and Cobalt linear accelerator (ViewRay) may have promising potential for brain tumors because of the imaging quality but needs to be confirmed in future clinical trials.

## Clinical Studies Demonstrating the Potential Role of IGRT in the Treatment of GBM

The combination of better tumor delineation, daily treatment imaging, and sharp dose gradient makes IGRT an ideal tool for radiotherapy because of the potential for dose escalation and reduced toxicity to the normal brain compared to 3-dimensional conformal radiotherapy (3D-CRT). As most studies failed to demonstrate an improvement in local control and survival in GBM patients with radiation dose escalation because of the tumor radio-resistance, accelerated radiation treatment may provide the patient with a better QOL and more quality time with their loved ones if the shortened treatment is equally effective compared to the conventional fractionation (30 days of radiotherapy). Preliminary studies of intensity-modulated radiotherapy (IMRT) for GBM demonstrated the feasibility of this treatment alternative. A total of 24 patients with resected GBM received postoperative hypo-fractionated IMRT to the surgical cavity and residual tumor to 60 Gy in 10 fractions (6 Gy/fraction) and concurrent TMZ. The median survival was 16 months comparable to historic control in patients treated with conventional fractionation (36). Other studies also corroborated the efficacy and safety of hypo-fractionated IMRT for GBM (37). Compared to IMRT, IGRT may allow for reduction of the planning target volume because of daily pre-treatment imaging and the accuracy of the technique. Thus, IGRT is particularly useful when the tumor is located close to critical radiosensitive structures such as the optic chiasm, optic nerves, and brain stem. IGRT has been used as a boost dose for these indications to deliver a high dose to the tumor bed without increasing the risk of complications (38). Patients with GBM close to radiosensitive structures can also be treated with IGRT through the whole course of treatment with the SIB technique delivering a higher dose to the tumor (66 Gy instead of 60 Gy) without any complications (39). The course of radiotherapy can also be reduced to three to six treatments with IGRT without excessive toxicity (40). Other studies also corroborated the efficacy and safety of hypo-fractionated IGRT for GBM with fractionation ranging from one to eight treatments (41, 42).

The best illustration of the indication for IGRT in the treatment of GBM may be its role in the re-irradiation of recurrent tumor following standard chemoradiotherapy. Depending on tumor size, a single or multiple fractions may be delivered with IGRT for salvage. The steep dose gradient between the tumor and surrounding tissues decreases the risk of brain radio-necrosis. Median survival following salvage IGRT ranges from 7 to 11 months with or without chemotherapy (43–47). Toxicity of IGRT for re-irradiation remains acceptable. As most IGRT studies for recurrent GBM were based on MRI for tumor delineation, it would be interesting to see if integrating FET-PET into radiotherapy planning would improve local control and survival. A preliminary study PET study using ^11^C Methionine, a radiolabeled amino acid with a shorter half-life compared to FET, suggests that the median survival of patients with recurrent GBM undergoing molecular imaging for radiotherapy planning is superior to the ones of patients who had conventional MRI (48). Median survival was respectively 9 and 5 months for IGRT with and without biological imaging. The data is intriguing and merits further investigation.

## Clinical Studies Demonstrating the Potential Role of IGRT in the Treatment of Brain Metastasis

As survival of patients with brain metastasis depends on the control of systemic disease, it would be logical to provide radiotherapy within a short time frame to avoid delay in initiating chemotherapy. Radiosurgery would be one option because treatment would be delivered in one fraction. The caveat of radiosurgery is the high risk of recurrence in the non-treated areas of the brain. Adding WBRT may decrease the risk of recurrence in other areas of the normal brain but may worsen neurocognitive function and delay chemotherapy. The ideal treatment for brain metastases would be a combination of high radiation to the tumor, a reasonable treatment time to allow chemotherapy initiation, and preservation of neurocognitive function if feasible.

Preliminary studies of whole brain IGRT with SIB to the brain metastases have been very encouraging. A phase I study of 48 patients with one to three brain metastases reported no increased toxicity when the whole brain was treated to 30 Gy in 3 Gy/fraction while the brain metastases were treated on a dose escalation schedule ranging from 35 Gy (3.5 Gy/fraction) to 60 Gy (6 Gy/fraction). Only 8 out of 48 patients (14%) developed progressive disease in the brain (49). A later pooled analysis of 120 patients with brain metastasis confirmed the safety of this approach. Seventy patients with one to three brain metastases were treated according to the previous protocol of 30 Gy in 3 Gy/fraction to the whole brain and 50 patients with one to six brain metastases were treated to 20 Gy in 4 Gy/fraction to the whole brain and 40 Gy in 8 Gy/fraction to the brain metastases (50). Twenty-one patients (23%) died from intracranial disease progression. Three patients developed tumor necrosis but there was no death from treatment toxicity. Thus, whole brain IGRT with SIB seemed to achieve good local control for patients with one to six brain metastases within 1–2 weeks of radiotherapy. The absence of toxicity of whole brain IGRT with SIB was also corroborated in another study. Twenty-nine patients with one to four brain metastases were treated to 30 Gy in 3 Gy/fraction to the whole brain and 40 Gy in 4 Gy/fraction to the brain metastases (51). QOL and neurocognitive function were also tested. Three patients (13%) developed local failures. There was no impairment of neurocognitive function but QOL deteriorated 3 months after treatment. The cause of death in all three IGRT whole brain studies were predominantly systemic disease progression emphasizing the need for systemic disease control in patients with brain metastasis.

To protect long-term survivors from neurocognitive dysfunction following WBRT, sparing of the limbic system from excessive radiation should be considered. Technically, it is feasible to spare the hippocampus and NSCs compartment with IGRT without under-dosing the target volume (52, 53). Animal experiments demonstrated the feasibility of NSC sparing IMRT. Mice receiving NSC sparing IMRT developed less damage to the NSC compared to a non-sparing NSC technique (54). Thus, it would be interesting to combine whole brain IGRT with SIB and hippocampus sparing in patients with brain metastases to improve local control and preserve neurocognitive function in future clinical trials.

We emphasize that radiation dose escalation for brain tumors and brain metastasis should not be performed without appropriate image guidance because of the potential for increased neurotoxicity. Preliminary evidence suggests that when combined with advanced tumor imaging such as PET scan, IGRT may provide excellent loco-regional control while sparing normal organs from excessive radiation toxicity in patients with locally advanced head and neck cancer (55, 56). As an illustration, even a small organ such as the cochlea can be shielded from radiation when the gross neck nodes were treated to a curative dose of radiation (70 Gy). This may potentially decrease the risk of hearing loss (57). Similarly, IGRT, when applied incranially, may potentially maximizing normal neuro-tissue sparing, and potentially improves the patient’s QOL in patient with primary brain tumors and brain metastasis and needs to be investigated in future prospective studies.

## Conclusion

Image-guided radiotherapy is a promising technique to reduce treatment time in patients with GBM. In the future FET-PET may further improve treatment accuracy of IGRT and potentially improve local control. In patients with brain metastases, whole brain IGRT with SIB may allow improvement of local control and early initiation of systemic therapy for better survival. Sparing of the hippocampus with whole brain IGRT is intriguing and merits further investigation to preserve neurocognitive function. Prospective studies should be performed to investigate the feasibility of IGRT to improve QOL in patients with GBM or brain metastasis.

## Conflict of Interest Statement

The authors declare that the research was conducted in the absence of any commercial or financial relationships that could be construed as a potential conflict of interest.
